# IMRT/VMAT for malignancies in the head-and-neck region: Outcome in patients aged 80+

**DOI:** 10.1007/s00066-016-0986-8

**Published:** 2016-06-15

**Authors:** Michelle L. Brown, Christoph Glanzmann, Gerhard Huber, Marius Bredell, Tamara Rordorf, Gabriela Studer

**Affiliations:** Department of Radiation Oncology, Head Neck Cancer Center, University Hospital Zurich, Raemistrasse 100, 8091 Zurich, Switzerland; Department of Otorhinolaryngology, Head Neck Cancer Center, Head and Neck Surgery, University Hospital Zurich, Zurich, Switzerland; Department of Craniomaxillofacial and Oral Surgery, Head Neck Cancer Center, University Hospital Zurich, Zurich, Switzerland; Department of Medical Oncology, Head Neck Cancer Center, University Hospital Zurich, Zurich, Switzerland

**Keywords:** Elderly, Treatment outcome, Radiotherapy of elderly patients, Survival analysis, Radiation tolerance, Betagte, Behandlungserfolg, Radiotherapie im Alter, Überlebenszeitanalyse, Strahlentoleranz

## Abstract

**Objective:**

Elderly patients with malignant head-and-neck tumors (HNT) often pose a therapeutic challenge. They frequently have significant comorbidities which may influence their ability to tolerate tumor-specific therapies. Our aim was to investigate the outcome of patients aged 80+ years undergoing curative intent intensity- or volume-modulated radiation therapy (IMRT/VMAT).

**Methods:**

We retrospectively reviewed our HNT patients aged 80+ treated with curative IMRT/VMAT from December 2003 to November 2015. Overall survival (OS), disease-free survival (DFS), local control (LC), and treatment tolerance were assessed. Outcome results were compared with that of a younger HNT patient cohort from our hospital.

**Results:**

A total of 140 consecutive patients were included (65 postoperative, 75 definitive). Mean/median age at treatment start was 84.8/84.1 years (range 80–96 years). Mean/median follow-up time was 25/16 months (range 2–92 months). Of the 140 patients, 80 were alive with no evidence of disease when last seen, 28 had died due to the cancer, 12 remained alive with disease, the remaining 20 died intercurrently. Systemic concomitant therapy was administered in 7 %. Late grade 3–4 toxicity was observed in 2 %. All patients completed treatment. Hospitalization and feeding tube rates were 26 % and 11 %, respectively. The 2‑/3-year LC, DFS, and OS rates for the entire cohort were 81/80 %, 69/63 %, and 68/66 %, respectively. Squamous cell carcinoma (SCC) patients showed an inferior 3-year OS rate as compared to non-SCC patients (62 % vs 77 %, *p* = 0.0002), while LC and DFS did not differ. Patients undergoing postoperative radiation attained a higher OS compared to the definitively irradiated subgroup with 74 vs. 60 % at 3 years (*p* = 0.01); however, DFS rates were similar for both groups (68 vs. 61 %, *p* = 0.15). Corresponding rates for >1400 HNT patients <80 years treated during the same time interval were 81/80 %, 69/67 %, and 77/72 %, respectively.

**Conclusions:**

Treatment tolerance in our patients aged 80+ was high. These results suggest that elderly HNT patients should not be denied potentially curative treatment strategies.

## Zusammenfassung

**Ziele:**

Das therapeutische Vorgehen bei betagten Patienten mit Kopf-Hals-Tumoren (KHT) ist anspruchsvoll. Meist besteht eine relevante Komorbidität, was die Behandlungstoleranz beeinflussen kann. Ziel war es, Therapieerfolg und -toleranz unserer kurativ intensitäts- oder volumenmoduliert radiotherapierten (IMRT/VMAT) Patienten im Alter von >80 Jahren zu evaluieren.

**Methoden:**

Zwischen Dezember 2003 und November 2015 an unserer Klinik kurativ IMRT/VMAT-behandelte KHT-Patienten im Alter von >80 Jahren wurden retrospektiv analysiert. Gemessen wurden Gesamtüberleben (GÜ), krankheitsfreies Überleben (KFÜ), lokale Kontrolle (LK) und Therapietoleranz. Die Daten wurden mit unserer jüngeren Patienten-Kohorte verglichen.

**Resultate:**

Erfasst wurden 140 Patienten (65 postoperativ, 75 definitiv bestrahlt). Das mittlere/mediane Alter bei Behandlungsbeginn betrug 84,8/84,1 Jahre (80–96), die mittlere/mediane Verlaufsbeobachtungszeit 25/16 Monate (2–92). Bei Letztkontakt waren von den 140 Patienten 80 Patienten krankheitsfrei am Leben, 28 verstarben am Tumorleiden, 12 lebten mit Krankheitsmanifestationen, 20 verstarben interkurrent. Eine systemische Therapie wurde in 7 % simultan appliziert, Spättoxizität Grad 3–4 fand sich bei 2 %. Alle Patienten konnten die Bestrahlung abschließen; die Hospitalisierungs- bzw. Magensondenrate betrug 26 bzw. 11 %. Die 2‑/3-Jahresraten für LK, KFÜ und GÜ lagen bei 81/80 %, 69/63 % und 68/66 %. Patienten mit Plattenepithelkarzinomen (SCC) zeigten bei gleicher LK und KFÜ ein niedrigeres GÜ als jene mit Non-SCC (62 vs. 77 %; *p *=0,0002). Patienten nach postoperativer IMRT wiesen ein höheres GÜ auf als jene nach definitiver IMRT (74 vs. 60 % nach 3 Jahren; *p *= 0,01); das KFÜ war jedoch ähnlich für beide Untergruppen (68 vs. 61 %; *p *= 0,15). Die korrespondierenden Raten für unsere >1400 IMRT/VMAT-KHT-Patienten <80 Jahren betrugen 81/80 %, 69/67 % und 77/72 %.

**Fazit:**

Die Behandlungstoleranz unserer Patienten im Alter >80 war hoch. Dieses Ergebnis spricht dafür, betagten Patienten ein potenziell kuratives Vorgehen nicht vorzuenthalten.

## Introduction

Elderly patients with malignant head-and-neck tumors (HNT) pose a therapeutic challenge. They are often ineligible for systemic therapy and participation in clinical trials due to their advanced age and may have significant comorbidities that influence their ability to tolerate tumor-specific therapies. Other biological and social factors, such as limited mobility and social supports, can further impact the delivery of optimal oncological treatment. Radiotherapy plays a pivotal role in the management of HNT, particularly in those patients unable to undergo primary surgical treatment. Chemotherapy may be combined with radiation in cases of locally advanced disease with no contraindications to systemic treatment; however, the role of chemotherapy in elderly patients remains controversial as the Meta-Analysis of Chemotherapy in Head and Neck Cancer (MACH-NC) demonstrated a diminishing effect on survival with increasing age [1].

HNT is typically a cancer of the elderly population and U.S. data demonstrates that 47 % of mucosal HNT patients diagnosed between 1973 and 2008 were aged greater than 65 years [2]. In Switzerland, the life expectancy for women and men in 2009 was 84.4 years and 79.8 years, respectively, one of the highest in the developed world. The percentage aged greater than 65 years was 17 % in 2009 and is projected to reach 28 % by 2050 [3]. This projected increase in the number of elderly patients will result in an increased number of cancer diagnoses, as age is a known risk factor for cancer development. Defining “elderly” remains problematic and is often based on chronological age alone. The National Institute on Aging and National Institutes of Health have defined 3 categories: young old (65–74 years), older old (75–85 years) and oldest old (>85 years) [4]. Although such definitions are helpful to subdivide chronological age, the impact of comorbidities, quality of life, and functional status is not captured in such definitions. It is prudent to consider and improve upon methods by which elderly patients will be assessed for curative therapies [5] and to determine the outcomes for treatment in order to provide adequate and accurate information to patients.

The optimal radiotherapeutic management of elderly HNT patients cannot be easily defined at present, due to the paucity of randomized data, particularly of very elderly patients. There is underrepresentation of the elderly in HNT clinical trials, where the age limit is often restricted to 70–75 years [5]. Extrapolation of outcome from pre-existing data from the 3‑dimensional (3D) treatment planning era should be undertaken with caution, as contemporary management utilizes intensity-modulated radiotherapy (IMRT) or volume-modulated arc therapy (VMAT) which may confer a more advantageous toxicity profile as demonstrated in clinical trials [6].

To evaluate our institutional experience, we assessed the patterns of care, tumor control and treatment tolerance for patients 80+ undergoing curative radiotherapy for mucosal or nonmucosal HNT with IMRT or VMAT.

## Materials and methods

### Patient and treatment parameters

We retrospectively reviewed our HNT IMRT/VMAT database to identify patients with mucosal or nonmucosal HNT aged 80+ receiving curative radiotherapy from December 2003 to November 2015. All patients were initially discussed in our weekly interdisciplinary head and neck tumor board. Two multimorbid patients selected for curative radiation were excluded from this analysis: in one patient, IMRT was prematurely interrupted (3 fractions of 2 Gy) due to a sacrum fracture following a fall; a second patient developed cardiopulmonary decompensation following surgery prior to the start of radiation and did not sufficiently recover to commence curative radiation (2 of 142 patients with intention to treat).

Patterns of care, including treatment modalities received (surgery, radiotherapy, chemotherapy, immunotherapy) and ambulant or inpatient treatment were evaluated. Further patient, tumor and treatment characteristics were collected, including age, ECOG performance status (PS) at the time of initial radiation oncology consultation, radiotherapy dose, and fractionation. For comparison reasons, we used outcome data from our younger IMRT patients (<80 years) that had been collected in our database. Approval from the local ethics committee was obtained for data evaluation of our IMRT/VMAT cohort.

### Radiotherapy

Patients were immobilized in the supine position from the vertex to shoulders with commercially available thermoplastic masks. Planning CT images of 2‑mm slice thickness were acquired from the supraorbital ridge or vertex to the carina with iodinated contrast medium in all eligible patients. Our approach to target volume definition has been extensively outlined in a prior publication [7]. No specific modifications of our planning target volume (PTV) concepts were performed, except in a few patients with very large gross tumor volume (GTV), whereby the GTV was individually defined as PTV70 Gy, with a margin of approximately 0.5 to 1 cm defined as PTV68 Gy. These PTV adjustments were also used in younger patients and were thus independent of age.

Treatment plans were calculated using the Varian Treatment Planning System (Eclipse* External Beam Planning System, Version 7.3.10 and PRO 8.9, AAA 8.9, Varian Medical Systems). An extended field simultaneous integrated boost (SIB)-IMRT technique, whereby the primary tumor and regional lymph nodes are covered in a single phase by a 6-MV dynamic MLC system (Varian Medical Systems, Palo Alto, CA, USA) using a sliding window technique, or alternatively a VMAT technique, was used [7]. Our internal standard radiotherapy schedules were used (Table 1).

**Table 1:** Treatment-related parameters

Parameters	*n* (%)
*IMRT/VMAT techniques*	140 (100)
Definitive radiation	75 (54)
Postoperative radiation	65 (46)
*Treatment schedules*	
30–33× 2 Gy = 60–66 Gy (postop)	59 (42)
33× 2.11 Gy = 69.6 Gy (definitive)	29 (21)
35× 2 Gy = 70 Gy (definitive)	22 (16)
34× 2 Gy = 68 Gy (definitive, 6 fractions per week)	10 (7)
30× 2.2 Gy = 66 Gy (definitive)	3 (2)
2.5–3.5 Gy/fraction to 39–56 Gy	17 (12)
*Treatment delivery*	
Ambulatory	104 (74)
Inpatient	36 (26)
*Systemic therapy*	8/140 (5)
Carboplatin	1 (6 cycles)
Cetuximab	7 (5–7 cycles)
*Feeding tube *(hospitalization: included in the 36 inpatients listed above)	15 (11)

### Systemic concomitant therapy

Carboplatin was administered at AUC 2 when combined with radiotherapy. Adjuvant chemotherapy consisted of carboplatin AUC 5 and 5‑fluorouracil (5-FU) 1000 mg/m^2^ (6 cycles) and was given to one patient. Cetuximab was administered at standard doses of 400 mg/m^2^ loading dose, followed by 250 mg/m^2^ weekly during the course of radiotherapy. No cisplatin-based systemic therapy was given in this elderly patient subgroup.

### Follow-up

Patients were seen weekly during the course of radiotherapy, or more frequently as required. Weekly clinical reviews in the Department of Radiation Oncology were continued until the acute treatment toxicities had significantly improved or resolved. Patients were reviewed 4–6 weeks after completion of radiotherapy in our joint clinics at the Department of Head and Neck or at the Department of Maxillofacial Surgery. Our standardized follow-up program has been reported in prior publications [7], although frequency of follow-up was reduced in some patients in the current cohort due to age, limited mobility, or significant comorbid factors.

### Outcome

Tumor control was assessed in terms of overall survival (OS), disease-free survival (DFS), and local control (LC). Treatment tolerance: Hospitalization was defined as any admission to hospital during radiotherapy treatment. Placement of a feeding tube is routinely performed as an inpatient at our center and these patients were therefore included in the hospitalized group. Acute toxicity was not documented in detail in all patients; however, the need for hospitalization and the duration of radiation therapy (total treatment time vs. scheduled treatment time) were considered surrogate parameters for early treatment tolerance. Worst grade of late toxicity was scored according to the Radiation Therapy Oncology Group (RTOG)/European Organization for Research and Treatment of Cancer (EORTC) radiation morbidity criteria.

Detailed treatment toxicity data were not analyzed for the younger IMRT cohort in the statistical analysis; therefore, no comparison was performed with the elderly cohort.

### Statistics

Kaplan–Meier survival curves were performed using the StatView® (Version 4.5) statistics program. *P* values <0.05 were considered statistically significant.

## Results

### Patient and tumor characteristics

A total of 140 patients aged 80+ years out of 1606 (9 %) consecutively curatively treated patients with mucosal (98/140) and nonmucosal HNT were identified from our database and included in this analysis (Table 2). All patients had mucosal tissue included in the planning target volume. Mean/median age at treatment start was 84.8/84.1 years (range 80–96 years). Mean/median follow-up time was 25/16 months (range 2–92 months).

**Table 2:** Patient and tumor characteristics

Parameters	*n* (%)
Patients	140
Gender	46 women : 94 men
Follow-up, mean/median (range)	25/16 months (2–92)
Age mean/median (range)	84.8/84.1 years (80–96)
80–85 years	85 (61)
>85–90 years	36 (26)
>90 years	19 (13)
*Pre-IMRT performance* *status*	
0	82 (59)
1	41 (29)
2	12 (9)
3	5 (3)
*Histology*	
Squamous cell carcinoma	93 (66)
Spinocellular carcinoma	17 (12)
Melanoma	7 (5)
Thyroid carcinoma	5 (3)
Merkel cell carcinoma	4 (3)
Non-Hodgkin lymphoma (NHL)	4 (3)
Basal cell carcinoma	2 (1)
Others	8 (5)
*Diagnosis*	
Oral cavity	24 (18)
Skin	24 (18)
Oropharynx	21 (15)
Larynx	20 (14)
Hypopharynx	10 (7)
Salivary glands	10 (7)
Nose	7 (5)
Paranasal sinus	6 (4)
Thyroid	6 (4)
Unknown primary tumor	5 (3)
Non-Hodgkin lymphoma (NHL)	
Others	4 (3)
*T stages* (without NHL)	
r0	11 (8)
1	11 (8)
2	31 (22)
3	17 (12)
4	37 (26)
Recurrence	23 (16)
*N stages* (without NHL)	
0 (N0/rN0)	65 (46%) (59/6)
1	18 (13)
2a	5 (3)
2b	25 (18)
2c	11 (8)
3	4 (3)
Recurrence	8 (5)

More than 60 % of all patients presented with advanced T3/4, and/or N2c/N3, or recurrent disease after previous surgery alone.

Data from our younger IMRT cohort aged <80 years was evaluated and compared with the elderly group. Squamous cell cancer (SCC) was diagnosed in 66 % of the elderly versus 84 % of the younger patients. Both the >80 and <80 year subgroups showed approximately 50 % T3/4 primary stage tumors, while the advanced nodal status (N2c/N3) was 20 % for younger patients and 11 % for the older cohort. IMRT was postoperatively delivered in 46 and 41 % of patients aged >80 and <80 years, respectively.

### Treatment tolerance

Of 140 patients, 36 (26 %) required part or all of their treatment as an inpatient (Table 1). A total of 15 (11 %) patients required a feeding tube during treatment, including 1 patient who received the tube prior to commencement of radiotherapy. The need for a feeding tube was not related to WHO PS (10 tubes in 82 PS 0 patients, 4 in 41 PS 1 patients and 1 in 17 PS 2 patients). The need for a feeding tube was inversely related to the age intervals: no tube was required in 19 patients >90 years, 7 tubes in 36 patients aged 85–90 years, 8 tubes in 85 patients aged 80–85 years.

All patients were able to complete treatment. Total treatment times were maintained according to the initial schedule in 136 of 140 patients (74 %); 4 patients had a treatment delay between 5 and 11 days. Late grade 3 and 4 toxicity was observed in 3 patients (1 patient with grade 2–3 osteoradionecrosis following tooth extraction post IMRT; 1 patient required a persisting feeding tube; 1 patient with grade 2–3 xerostomia).

### Disease control

**Fig. 1:**
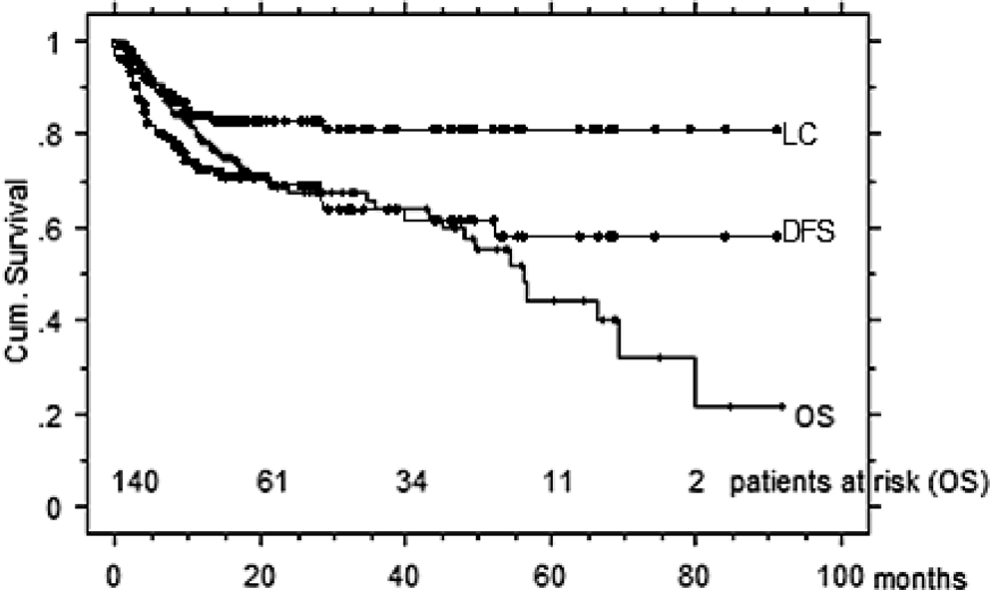
Disease control. *LC* local control, *DFS* disease-free survival, *OS* overall survival

**Fig. 2:**
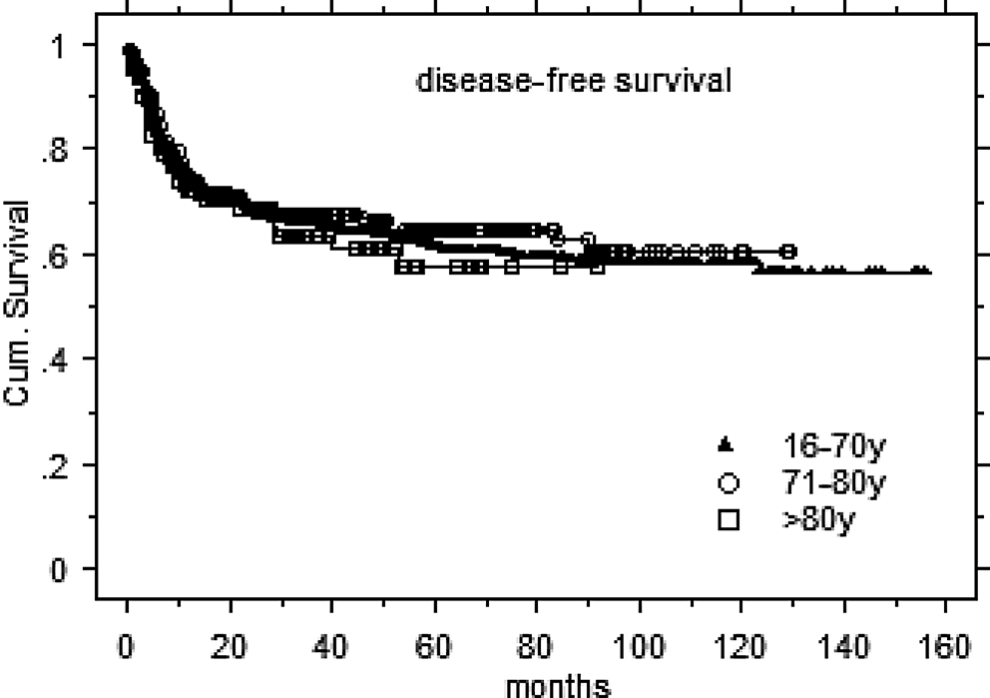
Disease-free survival related to age intervals. *y* years, *Cum. Survival* cumulative survival

**Fig. 3:**
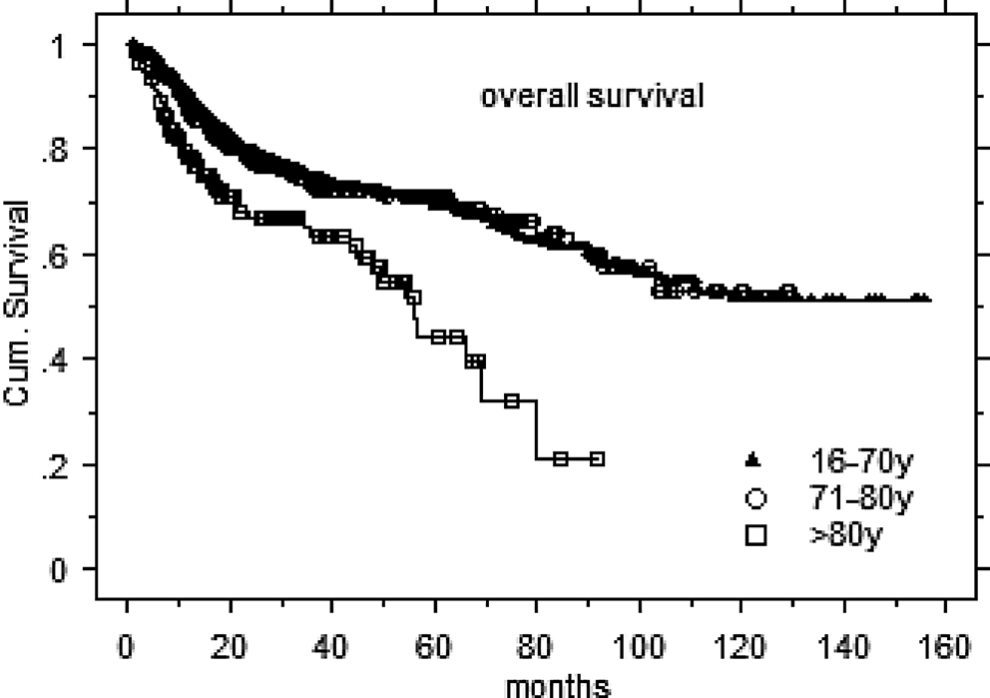
Overall survival rates related to age intervals. *y* years, *Cum. Survival* cumulative survival

The 2‑/3-year LC, DFS, and OS rates for the entire 80+ cohort were 81/80 , 69/63, and 68/66 %, respectively (Fig. 1). The corresponding survival rates for >1400 HNT IMRT patients aged <80 years (mean/median 60.9/62.7 years, range 16–79.8 years) and treated during the same time interval (with additional simultaneous systemic therapy in 71 %) at our center were 81/80 % (NS), 69/67 % (NS), and 77/72 % (*p* <0.001), respectively. DFS (Fig. 2) and LC rates (data not shown) of the 80+ cohort were comparable to our younger IMRT HNT patients; OS was inferior in the 80+ cohort (Fig. 3). Ambulatory elderly patients showed higher 3‑year OS than inpatients (approximately 70 vs approximately 50 %, *p* = 0.006). In addition, 80+ patients with pre-IMRT WHO performance status (PS) 0–1 demonstrated higher 3‑year OS than patients with PS 2–3 (approximately 70 vs approximately 40 %, *p *= 0.01). Gender did not impact on OS (*p* = 0.7) or DFS (*p *= 0.5). In addition, the need for a feeding tube did not result in any significant difference in OS (*p* = 0.8); however, the sample size of patients was unbalanced (*n* = 15 vs. 135 with and without a feeding tube, respectively). Patients undergoing postoperative radiation attained a higher OS compared to the definitively irradiated subgroup (fit enough for surgery and/or smaller tumors), with 74 vs. 60 % at 3 years (*p* = 0.01); however, DFS rates were similar for both groups (68 vs. 61 %, *p* = 0.15). SCC patients showed an inferior 3‑year OS rate as compared to non-SCC patients (62 vs. 77 %, *p* = 0.0002), while LC and DFS did not differ (NS).

## Discussion

We were motivated to determine the outcomes and treatment tolerance of very elderly patients with HNT treated with modern radiotherapy techniques. As this subgroup accounts for approximately 10 % of all patients referred for curative or postoperative IMRT or VMAT to our center, knowledge regarding tumor control and side effects is crucial. We chose an arbitrary age of 80+ years, as this was an age whereby treatment decisions were often challenging for our interdisciplinary team.

This is, to our knowledge, the largest published group of “older and oldest old” HNT patients treated with modern radiation techniques (Table 3; [8–15]). We found DFS and LC rates (data not shown) comparable to that of younger patients, and a high treatment tolerance. Treatment decisions made at our weekly interdisciplinary head and neck cancer center tumor board represent a selection process in favor of fitter patients, as patients considered too ill or noncompliant to undergo treatment with potentially substantial side effects were not selected for curative radiation. Regarding the decision for (or against) treatment, we could not draw any reliable decision-making assistance from the initial WHO PS or the need for a feeding tube.

**Table 3:** Radiotherapy only studies in elderly head-and-neck tumor (*HNT*) patients

Author, year [ref]	Treatment interval	Study type	Patient (No.)	Age (years)	Dose/schedule	Technique	Outcome	Toxicity	Completion rate
Lusinchi et al., 1990 [8]	1978–1983	Retrospective single institution	331 (277 curative)	>70	65–75 Gy in 2 Gy 5x/week or 2.5 Gy 4x/week	Non-IMRT	71 % LC in curative intent arm 5-year 21 and 0 % for age 80–85 and over 84 years	Acute: severe epithelitis 1 %, Severe mucositis 17 % Late: mucosal necrosis 3.6 % Osteonecrosis 2.1 %, Laryngeal dyspnoea 2.7 %	91 % curative
Huguenin et al., 1996 [9]	1996	Retrospective single institution	75	≥75	70 Gy in 1.8–2 Gy 74.4 Gy in 1.2 Gy 2x day	3D CRT	5-year OS 30 % 2-year LC 83 % T1–2 N0, 39 % for advanced disease	Acute: NA Late: 1 bone necrosis	92 %
Pignon et al., 1996 [10]	1980–1995	Prospective EORTC trial database	1589	20–82 12 % >70	4 trials compared conventional fractionation to multifractionation; 1 trial compared CT plus surgery and RT to surgery and RT alone	Non-IMRT	No difference in OS or LRC Between age groups	Acute: functional mucosal reaction G3 and G4 more frequent in older ages, effect no longer significant when PS was controlled for Late: no difference between age groups	NA
Zachariah et al., 1997 [11]	1988–1995	Retrospective, 2 institutions	35 (included brain and upper aerodigestive tract tumors)	≥80	50–77.8 Gy in 1.1–2.5 Gy	Non-IMRT	CR 66 %	Acute: 1 G4 mucositis Late: NA	NA
Mitsuhashi et al., 1999 [12]	1970–1997	Retrospective single institution	14 (11/14 curative)	≥90	Median curative dose 61.2 Gy (35–78)	Conventional EBRT	RR 90 % CR 60 %	Acute: G3–4 mucositis 40 % Late: NA	91 % (curative cases)
Allal et al., 2000 [13]	1991–1997	Retrospective single institution	39	≥70	69.9 Gy in 41f over 38 days	Non-IMRT	3-year OS 68 % 3-year LRC 73 %	Acute: G3 64 % G4 2.6 % Late: RTOG G3–4 toxicity 3 %	100 %
Schofield et al., 2003 [14]	1991–1995	Retrospective single institution	98	≥80	50–55 Gy/16f 45–47.5 Gy/16f	Non-IMRT Beam directed technique	5-year OS 28 % 5-year CSS 59 % 5-year LC 70 %	Acute: NA Late:1 osteo-radionecrosis 1 oro-antral fistula 1 laryngeal perichondritis	98 %
Huang et al., 2011 [15]	2003–2007	Retrospective single institution	238	≥75 (7 ≥80y)	51 Gy/20f 60 Gy/25f 64 Gy/40 f, 2f/day 70 Gy/35 f, CRT 70 Gy/35 f, RT Other	Conformal or IMRT	1-year CSS 83 % 2-year CSS 71.7 % 5-year CSS 64.9 %	5.9 % ≥ G3 RTOG toxicity	96 %
**Own data, 2016**	2003–2015	Retrospective single institution	140	≥80	70 Gy/33–35f 66 Gy/33f 60 Gy/30f other	IMRT or VMAT	3-year LC 80 % 3-year DFS 63 % 3-year DFS 66 %	3 % G3 (1x Osteo-radionecrosis, 1x xerostomia, 1x feeding tube dependence)	100 %

*NA* not available, *LC* local control, *OS* overall survival, *CR* complete response, *RR* response rate, *CSS* cause-specific survival, *PS* performance status, *G* Grade, *RTOG* Radiation Therapy Oncology Group, *EORTC* European Organisation for the Research and Treatment of Cancer, *f* fractions

One of the limitations of our study was the inclusion of patients with nonmucosal sites. We acknowledge that treatment tolerance may be different in this group as less mucosa may have been included in the radiation field; however, the radiation dose applied was very similar. Patients treated for NHL (3 %) received significantly lower doses than mucosal HNT, but comprised a very small number of our cohort. Patients with spinocellular carcinoma underwent extensive lymphatic pathway irradiation and were not at substantially lower risk for side effects than patients with, for example, early stage laryngeal carcinoma. We included 7 patients with nasal tumors (including nasal mucosa only) as a dose of 70 Gy to the nose is not easy to tolerate at any age, which was our rationale for including these patients in our elderly cohort. As the aim of this analysis was to evaluate whether irradiating elderly HNT patients with curative intent is beneficial compared to its potential risk, we included all patients irrespective of mucosal or nonmucosal tumor origin.

We also included postoperatively irradiated patients, as the question of the benefit–risk ratio of postoperative radiation therapy is often difficult to decide in elderly patients. Although these patients are exposed to a somewhat lower radiation dose, they underwent and survived a surgical procedure; conversely, patients not fit enough for surgery receive a higher radiation dose and are, thus, exposed to other potential risks. The risk level of the two approaches are difficult to compare and may be dependent on the individual patient, their comorbidities, their treatment wishes, or the potential treatment bias of the treating physicians.

The study was also limited by the retrospective nature of the analysis. The limited follow-up of our patients can also be attributed to their age and other comorbid or social problems, which prevented their attendance for ongoing follow-up.

The total number of patients with theoretically curable malignant HNT who were not selected for curative treatment is not available to us, however is estimated to be low (<5 patients/year). In addition, having a diagnosis of cancer at an advanced age and being an older individual who is well enough to attend for a specialist consultation, may already represent a substantial positive preselection for curative treatment.

Consistent with our own cohort, many of the published series confirm a high rate of treatment completion in the elderly population (approximately 90–100 %) suggesting that patients either tolerate treatment well and/or were well selected to receive curative therapy. Data from a prospective study from Pignon et al. [10] noted similar rates of OS and locoregional control in patients less than or greater than 70 years. This could be confirmed by our results (Fig. 3), showing very similar OS rates for patients <70 and 70–80 years. Some of the published series show 5‑year OS rates of 20–30 %, reflecting the influence of reduced life expectancy in this group and the competing risk from comorbid conditions. Our data demonstrate improved 3‑year OS outcomes in comparison to the published data: approximately 70 % for patients less than 80 years, approximately 50 % for the subgroup 80+ (Fig. 3). We found only three further reports assessing patients 80+ (Zachariah et al., *n* = 35 [11], Mitsuhashi et al., *n* = 11 [12], Schofield et al., *n* = 98 [14]), therefore, limiting data comparison (Table 3).

There have been an increasing number of publications assessing the tolerance and outcome of radiochemotherapy regimens in elderly patients (Table 4; [16–23]). Nguyen et al. [21] evaluated the toxicity and outcomes of various chemotherapy regimens combined with modern radiotherapy techniques in patients aged younger and older than 70 years. They demonstrated no significant difference in grade 3 or 4 toxicity between the two groups and similar rates of tumor control. Machtay et al. [17] undertook a retrospective analysis of RTOG trial data with a focus on late toxicity. They demonstrated a significant increase in late severe toxicity for elderly patients, although there were only 27 patients aged greater than 70 included in these three studies. When combined with the subgroup analysis finding of decreasing effect of chemotherapy with age in the MACH-NC meta-analysis of chemoradiation trials, one needs to proceed with caution when offering combined modality therapy to elderly patients [1]. In our cohort, one very fit and motivated patient received chemotherapy, which was an exceptional case.

**Table 4:** Radiochemotherapy studies in elderly patients with head-and-neck tumor

Author, year [ref]	Date of treatment	Study type	Patient (No.)	Age	Dose	Chemotherapy type	Technique	Outcome	Toxicity	Completion rate
Kodaira et al., 2005 [16]	NA	Phase 1 prospective	11	≥70	60–70 Gy/30–35f or 39.6 Gy in 1.8Gyf re-RT	Weekly docetaxel, starting dose 10 mg/m^2^, additional increase of 2 mg/m^2^ up to maximum tolerated dose allowed	3D or dynamic rotation	See toxicity data	Acute: No G3 or higher hemtatological toxicity G3 mucositis in 6 pts, G4 in 3 pts Late: NA	NA
Machtay et al., 2008 [17]	1991–2001	Retrospective secondary analysis of RTOG trial late toxicity data	230	≤70 and >70 (*n* = 27)	Refer to following protocols: RTOG 91–11 RTOG 97–03 RTOG 99–14		Mostly 2D planning, no IMRT	See toxicity data	Acute: NA Late: Crude rate of late toxicity 43 %. Older patients significantly more likely to have severe late toxicity	NA
Koussis et al., 2008 [18]	1999–2002	Prospective phase II (included pat. with esophageal cancer)	35	≥70 (16 elderly)	70 Gy/35f	Neoadjuvant carboplatin and vinorelbine + concurrent carboplatin	No IMRT 3D conformal	2-year OS 41.5 %	Acute: 8.5 % grade 4 mucositis with febrile neutropenia Late:1 osteoradionecrosis	100 % for radiotherapy and concurrent carboplatin
Tsukuda et al., 2009 [19]	2002–2007	Prospective feasibility study	50	13 >75	66–70.2 Gy in 1.8–2 Gy	2 courses of S‑1 with RT – 50 or 40 mg bd, 2 weeks on, 1 week off	NA	2-year DSS 92 %, 2‑year OS 75 % Stage III and 38 and 29 % Stage IV	Acute: 18 % grade 3 hematological, 28 % nonhematological No grade 4 toxicity Late: NA	100 % for RT, 72 % for S1-administration
BoscoloRizzo et al., 2011 [20]	2000–2007	Prospective single institution	44	>65	66–70 Gy/33−35f	IC cisplatin 100 mg/m^2^ day 1, 5‑FU 1000 mg/m^2^ for 5 days Concurrent cisplatin 100 mg/m2 day 1/5-FU 1000 mg/m2 for 5 days week 1 and 4	3D conformal	3-year LRC 76.5 % 3-year PFS 67 % 3-year OS 70.9 %	Acute: 65.9 % G3–4 toxicity (G3–4 mucositis 34.1 %) Late: 29.5 % ≥Grade 3 late toxicity (3pts permanent tracheotomy, 4 permanent PEG)	84.1 % per protocol
Nguyen et al., 2012 [21]	2008–2011	Retrospective single institution	112, 27 ≥70 years	<70, ≥70	70 Gy/35f 66 Gy/33f	Cisplatin weeks 1,4,7 Carbo weekly Cisplatin weekly Carbo week 1,4, 7 5-FU and cisplatin Taxol	IMRT or IGRT	2-year OS 74 and 67.5 % for <70 and ≥70 respectively	Acute: 59.2 % G3–4 mucositis, 25.9 % G3–4 hematological toxicity in elderly. No significant difference in G3–4 between young and elderly groups Late: 16 % younger, NA for elderly	92 % <70 96 % ≥70 1 elderly patient due to death in week 1 of RT (fall)
Merlano et al., 2012 [22]	1997–2008	Retrospective single institution	317, 224 <65 93 ≥65	<65, ≥65	66–70 Gy/33–35f	Adjuvant concurrent cisplatin/RT Alternating CTRT Induction CT and CTRT cetuximab and RT	3D conformal	Younger patients have significantly longer survival than elderly	Elderly patients suffered from infections, in particular pneumonia, more frequently than young patients; no difference in other toxicities	92 % <65 87 % ≥65 (*p* = 0.20)
Michal et al., 2012 [23]	1989–2007	Retrospective single institution	181, 137 <70, 44 ≥70	<70, ≥70	68–74 Gy/1.8–2 Gy or 72–74.4 Gy/ 1.2 Gy 2x day	5-FU and cisplatin weeks 1 and 4 plus gefitinib for those in a clinical study	Non-IMRT	5-year OS 63 vs 49 % and 5‑year DSS 74 and 71 % in <70 vs. ≥70	More unplanned hospitalizations (84 vs. 67 %) and feeding tubes (89 vs. 69 %) in ≥70	NA

*NA* not available, *f* fractions, *re-RT* re-irradiation, *RT* radiotherapy, *IC* induction chemotherapy, *G1–4* grade 1–4, *OS* overall survival, *DSS* disease-specific survival, *LC* local control, *LRC* locoregional control, *5-FU* 5-fluorouracil

Immunotherapy is considered a viable option for those patients unable to receive systemic chemotherapy. We identified 7 of 140 patients who received between 5–7 cycles of concomitant cetuximab therapy in our patient cohort. The recent update of the Bonner et al. [24] study demonstrated on subgroup analysis that cetuximab had no benefit in patients aged 65 years or older; however, this analysis was underpowered due to small patient numbers aged greater than 65 years and age was not a study endpoint. Alongi et al. [25] reported on a phase II study in elderly patients utilizing cetuximab in combination with IMRT-SIB, which confirmed rates of toxicity consistent with the Bonner data [24].

A randomized controlled trial is currently assessing the impact of comprehensive geriatric assessment (CGA) on survival, function, and nutritional status in elderly HNC patients receiving standard care [26]. Whether these tools will impact on the appropriate selection of patients for curative radiotherapy is not yet known. Clearly there is a growing need for further research in the elderly oncological population. The International Society of Geriatric Oncology is addressing these issues and has undertaken a review of current best practice and priorities for research in radiation oncology for elderly patients [27].

## Conclusions

Treatment tolerance in our patient cohort aged 80+ was high. Local and disease-free survival rates were similar to that of younger HNT patients treated at our center. These results suggest that very elderly patients should be considered for potentially curative treatment strategies.
